# Oxidative Status of Marchigiana Beef Enriched in n-3 Fatty Acids and Vitamin E, Treated With a Blend of Oregano and Rosemary Essential Oils

**DOI:** 10.3389/fvets.2021.662079

**Published:** 2021-06-08

**Authors:** Isa Fusaro, Damiano Cavallini, Melania Giammarco, Anna Chiara Manetta, Maria Martuscelli, Ludovica Maria Eugenia Mammi, Lydia Lanzoni, Andrea Formigoni, Giorgio Vignola

**Affiliations:** ^1^Faculty of Veterinary Medicine, University of Teramo, Teramo, Italy; ^2^Faculty of Bioscience and Agro-Food and Environmental Technology, University of Teramo, Teramo, Italy; ^3^Department of Veterinary Medical Sciences, Alma Mater Studiorum University of Bologna, Bologna, Italy

**Keywords:** Marchigiana, muscle color, vitamin E, essential oils, meat, TBARS

## Abstract

The aim of this study was to evaluate the effects of basal dietary supplementation with linseed and linseed plus vitamin E in Marchigiana young bulls on the instrumental color, oxidative stability, and fatty acid composition of beef steaks with and without treatment with a blend of essential oils (oregano and rosemary) after storage times of 0, 3, 6, and 9 days. The fatty acid composition was evaluated in meat after 0 and 9 days of storage. No variation in the fatty acid composition of each type of meat was observed after storage. The use of dietary linseed and vitamin E, compared with linseed alone, significantly improved the oxidative stability, lightness (L^*^), and redness (a^*^) of the meat. A higher degree of oxidation was observed in meat samples from animals in the group fed linseed (L) followed by the control group (C) and the group fed linseed and vitamin E (LE). Moreover, the essential oils treatment (O) exerts a significant effect on beef oxidative stability of the group LE more than groups C and L during storage. The fatty acid composition of the meat was also affected by the diet: levels of healthful fatty acids (PUFA, n-3 PUFA, and CLA) were higher in meat from the groups that received linseed than the control group. Our results suggest that dietary vitamin E and treatment with essential oils are effective approaches to preserving the stability of beef cattle receiving a diet enriched in unsaturated fatty acids for up to 9 days of storage.

## Introduction

In Italy, the first meat that received the Protected Geographical Indication from the European Community was the “white beef of the Central Apennines” (1), which includes fresh meat from the three cattle breeds in central Italy: Chianina, Marchigiana, and Romagnola, which are, together with Piemontese, the main specialized breeds for meat production in Italy, and the quality of their products is widely recognized all over the world (2). In particular, Marchigiana husbandry techniques involve pasture in the summer and a stalling period during winter. In the finishing period, the animals are usually kept in stalls, and the fattening system is based on concentrates rich in corn and soybean meal, which improve the meat's fatty acid profile by conferring a high proportion of linoleic acid, thus increasing the n-6-to-n-3 ratio (3, 4).

The use of linseed to fatten bullocks has been demonstrated to be effective in increasing the percentage of n-3 fatty acids in the intramuscular fat (5). Recent years have seen considerable interest in the dietary supplementation of ruminants with polyunsaturated fatty acids (PUFAs), because of the positive effects of these molecules on human health, such as decreasing low-density lipoprotein and cholesterol, and conferring anti-inflammatory, anti-atherogenic, and anti-carcinogenic effects (6).

Several studies have reported favorable effects of dietary linseed supplementation on different ruminant products, such as milk, cheese, and meat (7–9). However, the natural enrichment of meat with PUFAs could potentially enhance meat's susceptibility to lipid oxidation and rancid taste (10), thereby altering meat sensorial properties and shelf life (11).

To overcome these adverse effects, a useful approach to improve meat oxidative stability and shelf life could involve endogenous or exogenous antioxidants. Consumers prefer antioxidants from natural rather than synthetic sources (12), because of the potential toxicity of the latter. Essential oils (EOs) are natural compounds extracted from officinal plants with antimicrobial and antioxidant properties; therefore, EOs have attracted interest as additives in the food industry (13). Experimental studies have shown that the antimicrobial potential is greater when a mixture of EOs, rather than single EO, is used (14, 15).

EOs from oregano (*Origanum vulgare* L.) and rosemary (*Rosmarinus officinalis* L.) have potential as natural food antioxidants (14, 16). Oregano and rosemary EOs are recognized as safe, and the final products meet consumer sensory acceptability requirements (17).

The aim of this study was to evaluate the effect of linseed and vitamin E dietary supplementation in Marchigiana beef cattle on the instrumental color, oxidative stability, and fatty acid composition of steaks with and without treatment with a blend of EOs from oregano and rosemary, after different storage times.

## Materials and Methods

### Animals and Diets

The Marchigiana young bulls were managed during the experiment according to the European Union (18), concerning the protection of animals kept for farming purposes, and were slaughtered according to the The Council of the European Union (19) of September 24, 2009, on the protection of animals at the time of killing.

The experiment was conducted at a commercial farm ~15 km northeast of Teramo, Abruzzo, Southern Italy. A total of 36 Marchigiana young bulls (440.7 ± 1.8 kg weight and 12 months of age) were allotted to three experimental groups according to body weight, such that the mean weight of each group was comparable.

Animals were stratified by weight and divided into three homogeneous groups that received the following diets during the finishing period of ~45 days: group C (control diet), group L (linseed), and group LE (linseed + vitamin E). Samples of total mixed ration (TMR) were collected every week and analyzed according to the standard methods of AOAC (20) for dry matter (DM), crude protein (CP), ether extract (EE), and ash. Neutral detergent fiber (NDF), acid detergent fiber (NDF), and acid detergent lignin (ADL) were determined as described by Van Soest et al. (21). All feed samples were also analyzed for FA composition as described by Lock et al. (22). The ingredients and chemical compositions of the diets are shown in Table 1. The dry matter intake was monitored by group and was calculated daily as the difference between the amount of feed offered and that refused. All animals were individually weighed every week to determine the average daily gain (ADG).

**Table 1:** Ingredients of the three experimental diets: C (control), L (linseed), and LE (linseed + vitamin E).

**Dietary ingredients**	**C**	**L**	**LE**
Dehydrated Alfa Hay (kg)	2.86	2.85	2.85
Straw (kg)	1.14	1.14	1.14
Corn meal (kg)	4.57	4.56	4.56
Extruded linseed (kg)	0.00	0.97	0.97
Beans (kg)	1.14	0.46	0.46
Cereal bran (kg)	2.29	2.28	2.28
Hydrogenate fat (kg)	0.29	0.00	0.00
Sodium chloride (kg)	0.06	0.06	0.06
Sodium bicarbonate (kg)	0.11	0.11	0.11
Vitamin E (g)	0.00	0.00	2.1
**Chemical composition (% DM)**			
Dry matter (%)	87.52	87.79	87.79
Crude fiber (%)	6.82	6.86	7.91
Crude protein (%)	13.87	13.91	13.91
Ether Extract (%)	6.84	6.84	7.06
Ash (%)	2.18	2.15	2.25
**Fatty acids composition (% total fatty acid)**			
SFA	51.16	14.84	16.72
MUFA	15.75	19.18	18.76
PUFA	33.09	65.98	64.52

### Carcass Traits

At the end of the finishing period, the young bulls were transported to an abattoir 30 min away. At the abattoir, the animals were weighed and slaughtered according to EU Regulations with a captive-bolt pistol and dressed according to commercial practice. The hot carcass weight (HCW) was recorded. The carcasses were graded for conformation and fatness by an expert evaluator according to the SEUROP classification (23). The SEUROP system defines six classes in order to classify carcasses according to their muscle conformation. The S class is “superior”; the E class is “excellent”; the U class is “very good”; the R class is “good”; the O class is “fair”; and the P class is “poor.” *M. longissimus dorsi* pH was measured *in situ* at 45 min *post mortem* (pH1) before entry into the cooling room, at the right side between the 5th and the 6th lumbar vertebrae (24), with a penetrating electrode adapted to a portable pHmeter (Crison pHmeter 507 and a 52–32 spear electrode, Crison Instruments, Spain). The carcasses were then cooled at 4°C for 24 h, and the final pH (pH24) was determined *in situ* with the same instruments on the same muscle. At 24 h, the carcasses were weighed (cold carcass weight). The dressing percentage was calculated as the ratio of the cold carcass weight to the final live weight. After 48 h in the cooling room, the carcasses were transferred to the laboratory for dissection under refrigerated conditions.

### Preparation of Steak Tray Samples

Eight steaks 25 mm thick from the left *longissimus dorsi* muscle between the last rib and the 6th lumbar vertebra of each carcass were aseptically cut and placed on a 17.5 × 25.5 × 4.0 cm polystyrene tray. Four steaks were treated by dipping into a blend of rosemary and oregano (1:1) EOs [0.5 ml rosemary + 0.5 ml oregano + 999 ml saline phosphate buffer (PBS) per kg of meat] for 60 s, and the rest were dipped in PBS solution as a control. PBS is isotonic and non-toxic. All samples (*n* = 288) underwent modified atmosphere packaging (66% O_2_; 25% CO_2_; 9% N_2_) and were then stored at +4°C for 9 days and sampled for the subsequent analysis.

Whole trays were placed in a dark chamber at 4°C and then removed from the chamber after 0, 3, 6, and 9 days for the following analyses: pH, instrumental color (lightness, L^*^; redness, a^*^; and yellowness, b^*^) and thiobarbituric acid reactive substances (TBARS). Ferric reducing antioxidant power (FRAP), vitamin E content and fatty acid profiles were determined at days 0 and 9 of storage.

### Meat Quality: pH and Color Measurements

The pH of the meat samples was measured with a penetrating electrode adapted to a portable pH meter (Crison pHmeter 507 and a 52–32 spear electrode, Crison Instruments, Spain). Steak samples from each treatment during storage (0, 3, 6, and 9 days) were subjected to pH recording and were allowed to bloom in direct contact with air for 1 h before colorimetric measurements. At the same time point, meat color parameters (lightness, L^*^; redness, a^*^; and yellowness, b^*^) were measured on steak samples according to the CIELab system with a Minolta Chroma Meter CR-300 (Minolta Camera Co., Osaka, Japan) with a D65 illuminant and an 8-mm aperture. Color measurements were performed on steak surfaces and are reported as the means of three measurements.

### Thiobarbituric Acid Reactive Substance Value

The lipid oxidation of meat samples was evaluated by TBARS measurement with the method of Inserra et al. (25) at 0, 3, 6, and 9 days of storage. Three replicates were run for each sample. Briefly, aliquots of 2.5 g of meat samples were homogenized with 12.5 ml of distilled water in a water/ice bath. Then, 12.5 ml of trichloroacetic acid (10% w/v) was added to precipitate proteins, and the samples were vortexed. The homogenates were filtered (Whatman No. 1), and 4 ml of filtrate was added to 1 ml of 0.06 M aqueous thiobarbituric acid in screw cap tubes. The tubes were kept in a water bath at 80°C for 90 min, and the absorbance of each sample was read at 532 nm with a UV spectrophotometer. TBARS values were calculated from a standard curve with different concentrations of TEP (1,1,3,3-tetraethoxypropane) and are expressed as milligrams of malonaldehyde (MDA)/kg of meat.

### Ferric Reducing Antioxidant Power Method

The spectrophotometric FRAP method was used for antioxidant capacity determination according to Szydłowska-Czerniak et al. (26). The FRAP reagent−2.5 ml of a 10 mmol/L TPTZ solution in 40 mmol/L HCl, 2.5 ml of 10 mmol/L FeCl_3_, and 25 ml of 0.1 mol/L acetate buffer (pH 3.6)—was prepared freshly and incubated at 40°C for 15 min. Then, 0.1–0.3 ml of methanolic extracts of meat samples and 2 ml of FRAP reagent were transferred into a 10-ml volumetric flask, and the volume was adjusted up to 10 ml with redistilled water. The blue solutions obtained were kept at room temperature for 10 min and centrifuged at 10,000 rpm for 10 min. The absorbance was measured at 593 nm against a reagent blank (2 ml of FRAP reagent adjusted to 10 ml with redistilled water). The results are expressed as μmol Trolox equivalent/g dw. Analyses were performed in triplicate on each extract.

### Vitamin E Analysis

Muscle vitamin E (α-tocopherol) concentrations were measured according to Franco et al. (27): 0.8 g of meat was minced and dissolved in 10 ml of methanol; then, internal standard (60 μg DL-α-tocopheryl acetate 98%) and 2 mg of BHT (butylhydroxytoluene) were added to avoid oxidation. The tube was vortexed for 30 s and then centrifuged at 4,000 g for 10 min at 20°C. Two milliliters of supernatant was removed and filtered through a 0.20-μm filter 13 mm in diameter. Aliquots of 10 μl were injected with an Alliance 2695 high-performance liquid chromatograph (Waters, Milan, Italy). A C18 (150 mm × 4.6 mm i.d., Atlantis®) column with a 5-μm particle size (Waters, Milan, Italy) was used. The mobile phase was HPLC-grade methanol acidified with 0.1% trifloracetic acid, at a flow rate of 1.4 ml/min. A 2,475 fluorescence detector (Waters, Milan, Italy) with 295 nm and 325 nm excitation and emission wavelengths, respectively, was used. Integration of peak areas of vitamin E was performed in Empower 2™ advanced software (Waters, Milan, Italy). The concentration of α-tocopherol was expressed as μg/g of fresh muscle. Standard solutions of α-tocopherol, 0.08–4.0 μg/ml, were prepared to obtain a calibration curve.

### Fatty Acid Profile

Briefly, intramuscular lipids were extracted according to the protocol of Folch et al. (28). After cold methylation of FAs with the technique of Frega and Lerker (29), the FA profile was determined at day 0 and 9 days of storage from extracts by gas chromatography with a Chrompack CP-SIL 88 capillary column (100 m length, 0.2 m film thickness, 0.25 mm inner diameter), from 160°C (1 min) to 175°C (4°C/min) for a total of 28 min, and then from 175 to 215°C (55°C/min) for 35 min with the injector at 250°C and the ionized flame detector at 260°C. Before statistical analysis, the data on FA composition were processed to calculate the following FA classes and indices: MUFAs (FAs with a single double bond), PUFAs (FAs with more than one double bond); SFAs (FAs without double bonds); n-3 (Σ n-3: sum of C18:3n-3, C20:5n-3, C22:5n-3 and C22:6n-3); and n-6 (Σn-6: sum of C18:2n-6t9,t12, C18:2n-6, CLAt10,c12, C18:3n-6, C20:3n-6, C20:4n-6 and C22:4n-6). The I-Harris index (30) was calculated as the sum of EPA and DHA, and the peroxidability index (PI) was calculated according to the following equation (31): (% dienoic acid × 1) + (% trienoic acid × 2) + (% tetraenoic acid × 3) + (% pentaenoic acid × 4) + (% hexaenoic acid × 5). FA quantities were expressed as mg FA/100 g sample.

### Statistical Analysis

Data were analyzed with GLM procedure of the SPPS software package for Windows (SPSS ver. 18; SPSS Inc., Chicago, USA), including the fixed effects of dietary treatment, use of essential oils and storage time and interactions between main effects. Results for interactions are not reported because they were not significant. Duncan's test was used to assess significant differences between means.

*P* < 0.05 was considered the threshold for significant differences. Results are presented as treatment mean and standard error of the mean.

## Results

### Animal Performance and Carcass Traits

The mean initial body weights were 441.9, 438.8, and 440.1 kg, and the mean final body weights were 516.0, 515.3, and 520.2 kg for C, L, and LE, respectively (Table 2). Dietary treatments did not affect the final live weights. The ADG of the animals was 1.66 kg/day for the animals in group C and 1.71 kg/day and 1.77 kg/day for the animals in group L and LE, respectively. Feed intake (kg/day) was similar among dietary groups (8.9, 9.1, and 9.3 kg/day for C, L, and LE, respectively).

**Table 2:** Effect of the experimental diets C (Control), L (Linseed), and LE (linseed + vitamin E) on Marchigiana beef *in vivo* performance.

	**Diet**			***P*-value**	**SEM**
	**C**	**L**	**LE**		
Initial weight (kg)	441.9	438.8	440.1	0.99	7.06
Final weight (kg)	516.0	515.3	520.2	0.93	8.25
Average daily gain (kg/day)	1.66	1.71	1.77	0.06	0.06
Slaughter age (days)	415.7	420.3	421.1	0.36	0.14
Feed intake (kg/head/day)^a^	8.9	9.1	9.3	0.98	0.25

a *Based on 12 animals/group*.

*SEM, Standard error of the mean*.

Carcass traits are presented in Table 3. The carcass weight, dressing percentage and carcass classification (conformation and fat cover) did not show differences among experimental groups. According to conformation, all carcasses were assigned to class U (very good). The dietary treatment did not influence pH1 and pH24. Nevertheless, the animals that received supplementation with linseed had a similar dressing percentage to that in the animals in group C.

**Table 3:** Effect of the experimental diets C (Control), L (Linseed), and LE (linseed + vitamin E) on Marchigiana beef carcass traits.

	**Diet**			***P*-value**	**SEM**
	**C**	**L**	**LE**		
Cold carcass weight (kg)	313.0	310.0	305.0	0.41	0.87
Dressing percentage (%)	61	62	61	0.87	0.98
pH1	6.40	6.35	6.38	0.33	0.45
pH24	5.54	5.48	5.48	0.33	0.16
SEUROP classification^a^	U	U	U	-	-
Fat classification (1–5)	3	3	3	0.89	0.12

a *The SEUROP system defines six classes in order to classify carcasses according to their muscle conformation. The S class is “superior”; the E class is “excellent”; the U class is “very good”; the R class is “good”; the O class is “fair”; and the P class is “poor”*.

*SEM, standard error of the mean*.

### Meat Quality Traits: pH and Color Parameters

**Figure 1:**
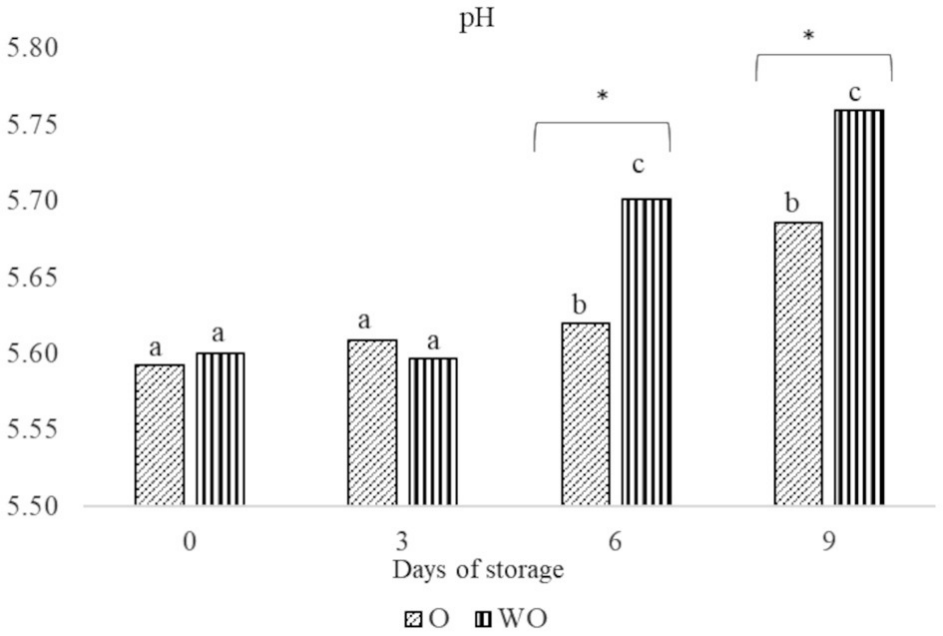
Effect of treatment with (O) and without (WO) essential oils on Marchigiana beef meat pH during storage time (0, 3, 6, and 9 days). Different letters on the top of the bars denote significant differences (*P* < 0.05) between experimental groups on days of storage. Asterisk denotes significant differences in the same group between the meat with (O) and without (WO) essential oils (*P* < 0.05).

The storage time and EO treatment significantly affected meat pH (Figure 1). The mean pH values were significantly lower in meat treated with EOs (O) after 6 and 9 days of storage (5.62 and 5.69, respectively) than in the samples without oils (WO) (5.70 and 5.76, respectively).

Table 4 presents the meat color parameters (L^*^, a^*^, and b^*^) of meat from three experimental groups with or without EO treatment. The L values decreased in all experimental groups, with storage time, and between the O and WO samples. In particular, in group C, the significant differences between the O and WO meat were evident from 3 days of storage, while in group L, it was evident at time 0. In contrast, in the LE group, we observed a significantly higher value of L^*^ in O meat than WO meat only at 9 days of storage. We observed the same trend for a^*^. Indeed, the a^*^ values decreased with storage time in all dietary treatments, and the values were significantly higher at time 0 and lower after 9 days of storage (*P* < 0.05). The meat treated with EO showed a higher value of a^*^ than the samples without EO at 6 (*P* < 0.05) and 9 days (*P* < 0.05) of storage in the C and L groups. In contrast, in the LE group, the redness value differed in O vs. WO meat samples only after 9 days of storage (*P* < 0.05). The b^*^ values showed significant differences among storage times. No differences were observed between the O and WO samples.

**Table 4:** Effects of time (0, 3, 6, and 9 days of storage), diets (C, L, and LE), and essential oils (O and WO) on Marchigiana beef meat color parameters (L^*^, a^*^, and b^*^).

**Parameters**	**Diet**	**EO**	**0**	**3**	**6**	**9**	**SEM**
L*		O	42.48a	43.63a	42.05b	41.82b	
	C			*	*	*	1.02
		WO	41.96a	41.17a	40.89b	40.91b	
		O	45.25a	45.31a	43.56b	42.85b	
	L		*	*	*	*	0.98
		WO	41.08a	40.28a	42.27b	41.04b	
		O	45.24a	44.72a	44.98a	43.74b	
	LE					*	0.87
		WO	44.95a	44.64a	43.10b	42.33b	
a*		O	18.54a	18.76a	17.15b	17.09b	
	C				*	*	0.25
		WO	18.09a	18.59a	16.36b	15.73c	
		O	18.81a	19.89a	18.97a	17.52b	
	L				*	*	0.56
		WO	19.59a	20.04a	17.61b	16.06c	
		O	22.91a	21.07a	21.46b	20.46b	
	LE					*	0.69
		WO	21.66a	21.03a	20.69b	19.09c	
b*		O	12.54a	12.04a	11.39a	10.76b	
	C						1.71
		WO	12.01a	12.05a	11.77a	10.48b	
		O	14.82a	14.01a	13.11b	13.19b	
	L						1.21
		WO	14.75a	13.9a	13.59b	13.35b	
		O	14.54a	14.60a	13.93b	13.58b	
	LE						0.95
		WO	14.51a	14.50a	13.10b	13.50b	

*C, Control; L, Linseed; LE, Linseed plus vitamin E*.

*WO, without essential oil; O, with essential oil*.

Asterisk in the column denotes significance in the same time point between meat treated with (O) or without essential oil (WO) *P < 0.05.

*Different letters on the same row denote significant differences (P < 0.05) between days of storage (0, 3, 6, and 9) in the same group*.

*SEM, Standard error of the mean*.

**Figure 2:**
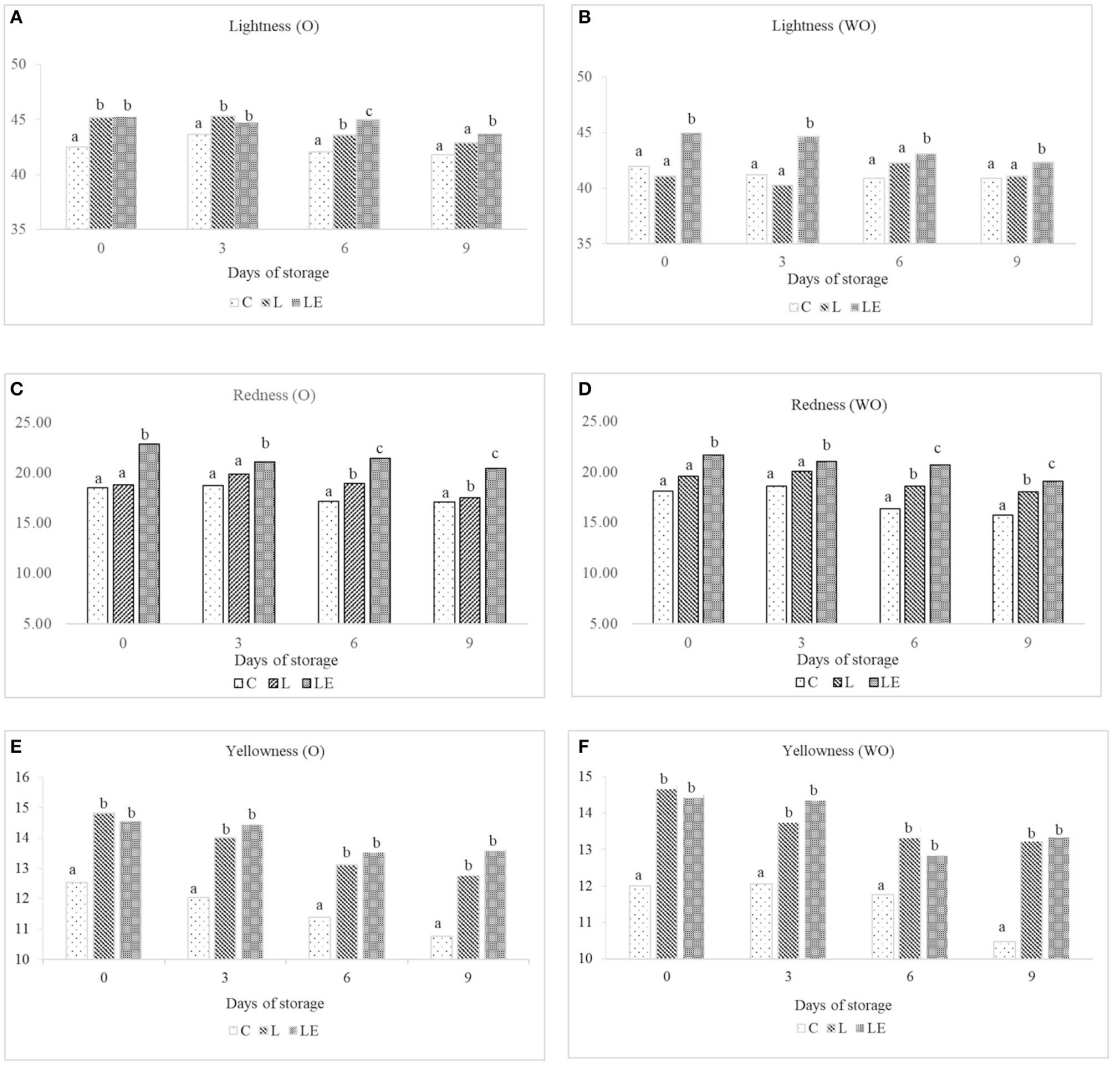
Effect of the experimental diets (C, L, and LE) on Lightness, L* **(A,B)** redness, a* **(C,D)**, and yellowness, b* **(E,F)** of Marchigiana beef meat treated with (O) and without essential oils (WO) during the storage time. C, Control; L, Linseed; LE, Linseed and vitamin E. Different letters on the top of the bars denote significant differences (*P* < 0.05) between groups during days of storage.

Figures 2A,B show the differences among the C, L, and LE groups at the same storage time. The lightness values were higher in the L and LE groups treated with O at 0 and 3 days of storage, than in the group C that showed the lowest value for each of these time points (*P* < 0.05). The lightness of C and L groups WO was lower for all the time points compared to group LE (*P* < 0.05). At 6 days of storage, we observed a significantly higher (*P* < 0.05) value of L^*^ in the LE group treated with EO than that in the C group, whereas L had an intermediate value. At 9 days, the L^*^ values were lower in C and L (*P* < 0.05) than in LE. The L^*^ values of WO meat samples were higher in the LE group than the L and C groups (*P* < 0.05) at all storage times.

The a^*^ (Figure 2C) differed significantly (*P* < 0.05) from days 0 to 9 between the experimental groups treated with EO. In particular, at time 0, a higher value of a^*^ was recorded for LE than C and L (*P* < 0.05). The same trend was observed on day 3. At 6 and 9 days of storage, a^*^ (*P* < 0.05) was significantly higher in LE than L, whereas the lowest value was recorded in C. We observed the same trend in WO meat samples (Figure 2D). The b^*^ values showed significant differences between dietary treatments at day 0 and during storage in L and LE compared with C (Figure 2E). The same trend was found in WO meat samples (Figure 2F).

### Meat Oxidative Stability

**Figure 3:**
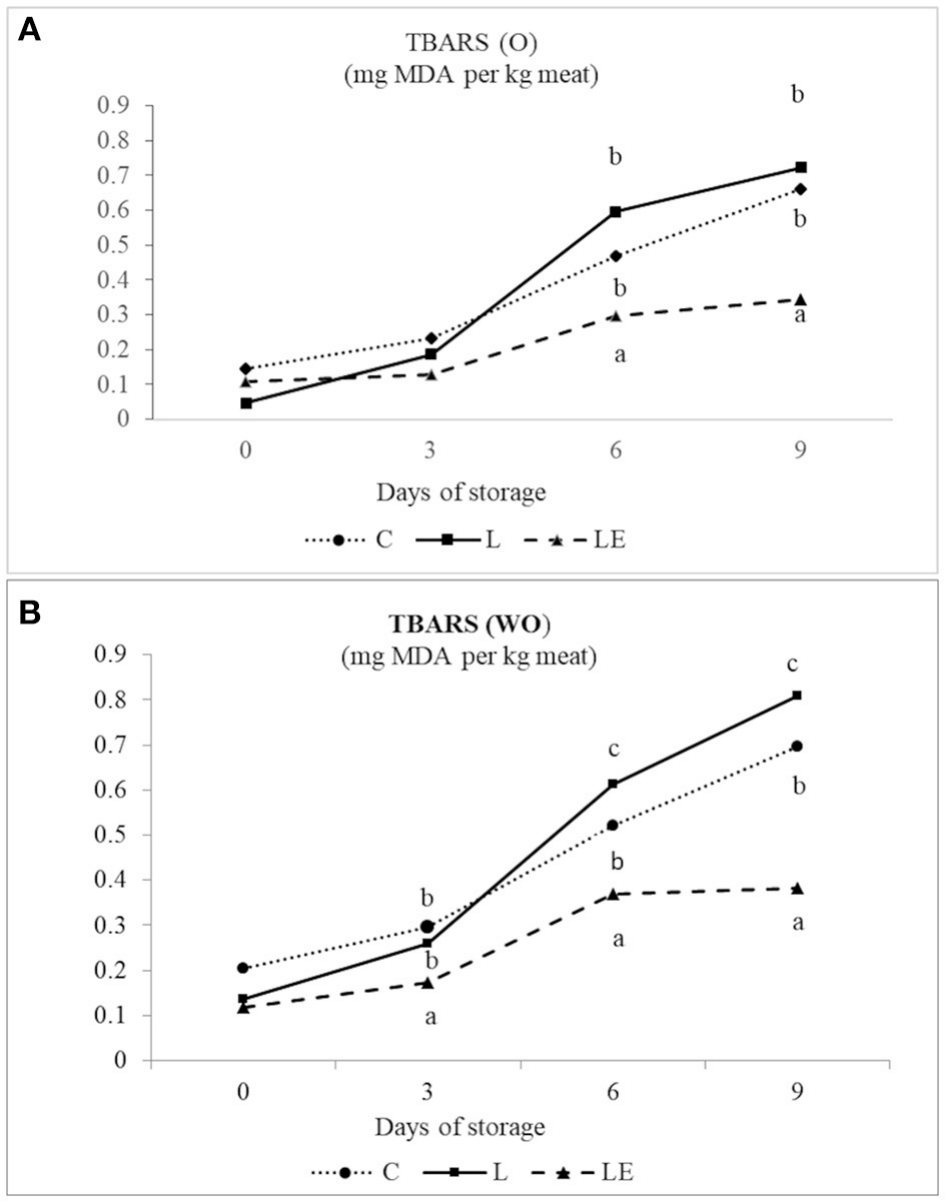
Effect of experimental diets (C, L, and LE) on TBARS concentration in Marchigiana beef meat treated with (O) **(A)** and without essential oils (WO) **(B)** during the storage time.

In O meat samples, no significant differences were found between dietary treatments for TBARS on days 0 and 3 of storage (Figure 3). On days 6 and 9 of storage, TBARS were significantly lower (*P* < 0.05) in the LE group (0.36 mg MDA/kg and 0.38 mg MDA/kg, respectively) than in the C and L groups (0.51 and 0.62 mg MDA/kg, respectively, on day 6; 0.72 and 0.80 mg MDA/kg, respectively, on day 9). In contrast, in WO meat samples, the TBARS values were significantly higher in C and L than LE from day 3. On days 6 and 9, the TBARS values were higher in L than C and LE (*P* < 0.05).

**Figure 4:**
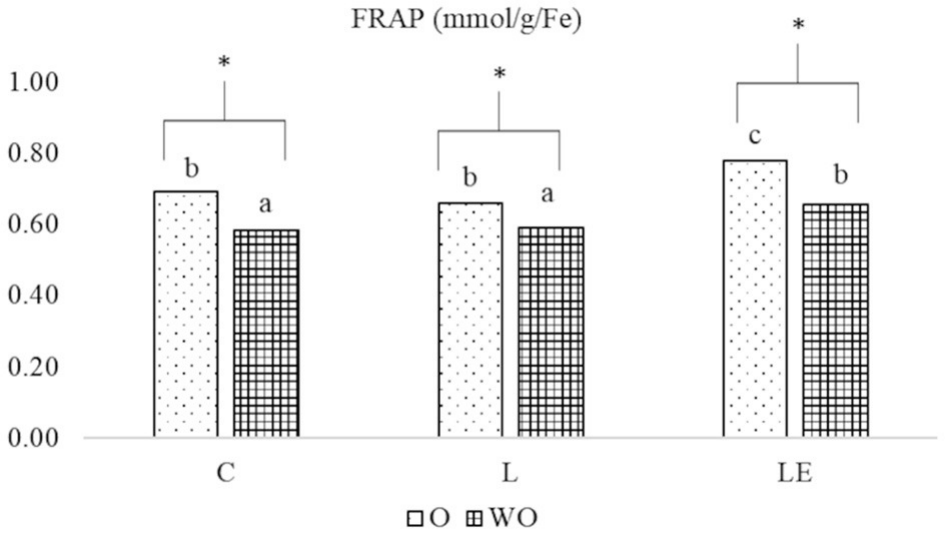
Effect of experimental diets (C, L, and LE) on FRAP concentration in Marchigiana beef meat treated with (O) and without essential oils (WO) at 9 days of storage. Different letters on the top of the bars denote significant differences (*P* < 0.05) between different dietary treatments (C, L, and LE). Asterisk denotes significant differences in the same group between the meat with (O) and without (WO) essential oils.

The FRAP values showed no differences between the dietary groups and for O and WO samples on day 0 of storage (data not shown). In contrast, on day 9, LE O samples had higher values than O and WO samples in the C and L groups (*P* < 0.05) (Figure 4). In all groups, the FRAP values were significantly different between the O and WO samples (*P* < 0.05).

**Figure 5:**
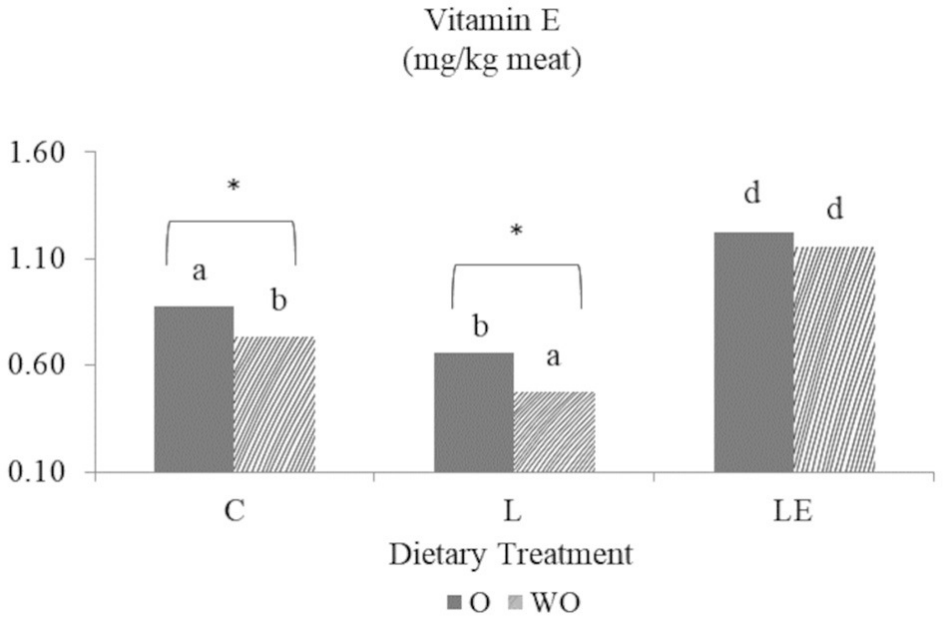
Effect of experimental diets (C, L, and LE) on Vitamin E concentration on Marchigiana beef meat treated with (O) and without essential oils (WO) at 9 days of storage. Different letters on top of bars denote significant differences (*P* < 0.05) between different dietary treatments (C, L, and LE). Asterisk denotes significant differences in the same group between the meat with (O) and without essential oils (WO).

The dietary content of vitamin E was significantly higher in the LE group (*P* < 0.01) than in the C and L groups at day 0 (data not shown) and at day 9 of storage (Figure 5). The use of EOs resulted in significantly higher vitamin E content in the C and L groups.

### Fatty Acid Profile

The FA composition of intramuscular fat is shown in Table 5. Dietary linseed supplementation significantly increased the content of PUFA (245.93 and 249.25 mg/100 g of sample, respectively, in L and LE, as compared with 201.94 mg/100 g of sample C), while SFA had an opposite trend, showing the higher value for group C (865.49 mg/100 g of sample) compared to L (753.87 mg/100 g of sample) and LE (742.18 mg/100 g of sample). L and LE also showed a higher content of n-3 PUFA than C, although these FAs were higher in LE (63.77 mg/100 g of sample) than L (47.41 mg/100 g of sample) (*P* < 0.05). The total n-6 PUFA was affected by the inclusion of dietary linseed, which were lower (*P* < 0.05) in L and LE than in C. The meat from L and LE, compared with C, had higher percentages (*P* < 0.05) of EPA and DHA (I-Harris index) (9.03 and 8.90 mg/100 g sample in L and LE, respectively, and 7.74 mg/100 g of sample in C). In contrast, the CLA value was higher in the LE group (14.47 mg/100 g of meat) and lower in the C group (5.02 mg/100 g of sample (*P* < 0.05), whereas the L group had an intermediate value (10.56 mg/100 g sample) between those of LE and C. In contrast, the PI was higher in LE than C, whereas L showed an intermediate value between those of C and LE (*P* < 0.05). On day 9, a significantly higher content of SFA was observed in WO meat, while PUFA, n-3, and n-6 were found higher in meat treated with O than WO meat samples.

**Table 5:** Effects of experimental diets (C, L, and LE) and treatment (EO) with (O) and without essential oils (WO) on health indices and pro-oxidant fatty acids on Marchigiana beef meat at 9 days of storage.

	**Diet**			**EO**		***P*****-value**		**SEM**
	**(mg/100 g of sample)**			**(mg/100 g of sample)**				
	**C**	**L**	**LE**	**O**	**WO**	**Diet**	**EO**	
SFA	865.49b	753.87a	742.18a	780.20b	790.68a	0.05	0.05	1.23
MUFA	720.96	698.52	707.89	732.69	712.15	0.35	0.87	0.98
PUFA	201.94a	245.93b	249.25b	238.63a	229.14b	0.05	0.05	0.56
n-3 PUFA	32.59a	47.41b	63.77c	50.60b	46.74a	0.05	0.05	1.32
n-6 PUFA	149.53b	118.37a	127.91a	135.36b	129.32a	0.05	0.0	1.75
n-6/n-3	4.58c	2.49b	2.00a	0.37	0.36	0.05	0.12	0.89
CLA	5.02A	10.56B	14.47C	11.02b	9.35a	0.01	0.05	1.85
PI	400.55a	408.31b	414.03c	405.04b	395.75a	0.05	0.05	1.87
I-HARRIS	7.74a	9.03b	8.90b	8.04	8.30	0.05	0.25	1.39

*The fat total content of meat (Longissimus dorsi muscle) from each diet was 2.29, 2.35, and 2.36% (on dry matter basis) for C, L, and LE respectively (P = 0.98)*.

*SFA, Saturated fatty acid; MUFA, Monounsaturated fatty acid; PUFA, Polyunsaturated fatty acid; CLA, Conjugated linoleic acid*.

*PI (Peroxidability index) = (% dienoic acid × 1) + (% trienoic acid × 2) + (% tetraenoic acid × 3) + (% pentaenoic acid × 4) + (% hexaenoic acid × 5)*.

*I-Harris = (EPA + DHA)*.

*Different letters in the same row indicate significant differences (a, b, c: P < 0.05; A, B, C P < 0.01)*.

*SEM, Standard error of the mean*.

## Discussion

### Animal Performance and Carcass Traits

The experimental diets did not significantly affect growth performance, in agreement with findings from other studies performed on finishing steers fed vegetable oilseeds (32–34). The lack of effect of linseed supplementation on animal performance may be ascribed to the similar energetic and protein content and to the similar dry matter intake. Generally, growth performance parameters depend primarily on breed, slaughter weight, age, and sex; because the animals were all of the same breed, age, and sex, it is plausible that there were no differences in growth performance (8). Similar results have been found by Kim et al. (35) in a study on the use of whole flaxseed in Hanwoo steers.

The ADG in the present study was slightly higher than the value observed by Mattii et al. (32) in Marchigiana beef (final weights ~755.0 kg); this effect may be ascribed to the different weights at slaughter.

Carcass traits did not show differences between treatment groups; in the present study, a lower dressing percentage was found than that reported by Pezzi et al. (36), probably because of the different ages and weights at slaughter. The dressing percentage increases with slaughter weight (8).

Carcass pH24 values showed no differences among the three experimental groups, ranging between 5.48 and 5.54; these values were within the normal range, as reported by Rousset and Renerre (37).

### Meat Quality Traits: pH and Color Parameters

The pH of meat packaged in a modified atmosphere in O and WO samples after storage under refrigerated temperature, is given in Figure 1. The significant increase in pH in all groups during storage may be attributed to microbial metabolism of secondary substrates after the depletion of glucose. According to Gill (38), after exhaustion of stored glucose, bacteria use amino acids released during protein breakdown, thus leading to the liberation of ammonia and amines, and consequently increasing the pH. Although the pH increased during storage, we observed significant differences between the O and WO samples. The O meat samples had a lower pH at days 6 and 9 of storage, probably because of the protective effect of EOs against substrate decomposition, as described by Ribeiro et al. (13).

Beef color parameters during the storage period indicated that color was influenced by the type of diet, the storage time, and the use of EOs.

As shown in Table 4, the L^*^ value decreased (*P* < 0.05) gradually in all experimental groups during storage and slightly differed depending on the presence or absence of EO treatment. In the group L, the differences between O and WO were evident from time 0, while from day 3 of storage, this difference was also evidenced in groups C and L, whereas the difference in the LE group was evident at 9 days of storage between the O and WO samples. Several studies (39–41) have reported higher L^*^ values for steak after supplementation of feedlot cattle with vitamin E and flaxseed rather than flaxseed alone. Our results suggest that the use of dietary vitamin E and the use of EO treatment of meat may protect the color stability of the meat more than the use of EOs alone. Our findings are also consistent with those of Arnold et al. (42), who confirmed that supplementation with α-tocopheryl acetate enhances color stability in beef, because α-tocopherol is closely associated with phospholipids in cell membranes (43). The L^*^ values varied significantly among the three dietary treatments, but with increasing storage time, the L^*^ decreased for all dietary treatments (Figures 2A,B). Other authors have reported that fat modification of meat from animals fed diets rich in oils is associated with high L^*^ of the finished product (44, 45).

Therefore, the presence of vitamin E in meat enriched with PUFAs increases stability against oxidation and makes the meat more desirable (46).

The a^*^ decreased with increasing storage time for all dietary treatments from day 0 to 9, and the lowest value was observed at day 9 of storage (Table 4). The discoloration of red meat cuts is generally associated with oxidation of the iron atom within the heme group in red oxymyoglobin to brownish metamyoglobin, and it depends on the presence of reducing systems and on lipid oxidation (11). Moreover, at 9 days of storage, the a^*^ value of LE was significantly higher in group O compared to WO. According to Zhang et al. (47), this result is probably due to the phenolic compounds of EOs, which can regenerate endogenous tocopherol in the phospholipid bilayers of lipoproteins. Moreover, the stabilizing effect of natural antioxidants on color has been described in other studies with various antioxidants from ingredients such as avocado (48) and rosemary and lemon balm (49).

In contrast, Juárez et al. (50) have reported that a dietary regimen with flaxseed and vitamin E did not preserve the color parameters in patties during storage. In the ground beef, in contrast to the steak used in the present research, cellular integrity is likely to be disrupted, owing to greater exposure of the tissues to oxygen and simultaneous dilution of antioxidant concentrations (51). The b^*^ was influenced by the storage time and the different dietary treatments (Table 4). The b^*^ value was higher in L and LE than C, at all storage times (Figures 2E,F). This finding agrees with results from Gomez et al. (45), who have found that the color parameters of meat, particularly yellowness, are closely associated with the color properties of the raw materials used in the diet formulation. In all groups on days 6 and 9, the b^*^ values had decreased significantly; in contrast, some authors (27, 52) have found that an increase in b^*^ reflects degradation of beef meat quality and is notably positively correlated with the sensory degradation of color, an increase in microbial count, fat oxidation, and other negative effects.

### Meat Oxidative Stability

To predict the oxidative stability of lipids in meat samples, we used TBARS tests. Values above 2 mg/kg of meat are considered critical, because they indicate a level of lipid oxidation products that can produce a rancid smell and taste that can be detected by consumers (53). Figures 3A,B show that the TBARS values were significantly influenced by the diet, storage time, and use of EOs. The results showed a higher degree of oxidation in meat samples from animals in the L group, followed by the C group and LE group. These results were evident for both O and WO samples. The use of EO treatment in meat samples in group L and LE resulted in lower lipid oxidation at day 6 than that in WO samples. The lipid oxidation increased in groups C and L after 3 days of storage. These results differ from those reported by Rojas and Brewer (54), in which beef patties treated with rosemary EO showed significantly higher TBARS content than controls on day 4 of storage. The presence of salt probably accelerated lipid oxidation. Moreover, minced meat develops high levels of oxidation, because fatty acids are exposed to pro-oxidants, such as iron released from muscle cells, and to the effects of greater bacterial degradation (55).

These results suggest that the use of EOs in beef steak may be a favorable strategy to limit the oxidation process in meat, owing to their high concentrations of phenolic compounds (56), carnosic acid, carnosol, rosmarinic acid, caffeic acid, and flavonoids that are responsible for antioxidant activity (57, 58).

Our results are in accordance with those of Juárez et al. (50), who have shown a decrease in TBARS concentrations after 3 days of storage in beef from animals fed flaxseeds and vitamin E, and a significant increase in these substances in meat obtained from animals supplemented exclusively with flax seeds.

The literature also highlights the combined effects of using rosemary and oregano EOs. Rosemary extract is highly antioxidant and therefore is widely used in the food industry (13). Several authors have reported the efficacy of rosemary in decreasing lipid oxidation in various foods (59, 60). Oregano extract is effective in inhibiting peroxidation through the initial neutralization of free radicals, subsequent blocking of peroxidation and finally chain-breaking activity (61).

Our FRAP results confirmed the findings of Berdahl and McKeague (58) and Rodríguez-Rojo et al. (60), who have reported a high ability of EOs to prevent oxidation. Moreover, the antioxidant capacity of meat treated with EOs remained high during storage, as compared with that of samples not treated with EO, thus indicating that the active antioxidant compounds of rosemary and oregano are able to maintain antioxidant activity over time.

Vitamin E content, as expected, was higher in LE and lower in L, whereas C showed an intermediate value. Because α-tocopherols rapidly neutralize oxidation (62), we presumed that the higher and easily oxidizable substrate (PUFA) content in L required a greater expenditure of vitamin E. Moreover, Dal Bosco et al. (63) demonstrated that meat from rabbits fed linseed required a higher expenditure of vitamin E, probably due to the ability of the vitamin E to rapidly counteract the oxidation as described by Burton and Traber (62). Vitamin E 2.1 g/head/day added to the diet of Marchigiana beef cattle at ~518.0 kg BW is adequate to obtain greater oxidative stability in the meat and consequently a longer shelf life of the product.

### Fatty Acids Profile

Results obtained at day 0 were compared with results obtained at 9 days to estimate the fatty acid profile evolution. Indeed, no variation in fatty acid profiles was observed after storage in all experimental groups (data not shown). These findings are consistent with those of Douny et al. (64), who have reported no differences in FA profiles in pork meat after 6 weeks of +4°C storage. The results of our study showed that the LE and L groups had similar concentrations of PUFAs, but the n-3 values were lower in L than LE meat samples. These results are probably due to the effect of vitamin E on the rumen microbiota. As suggested by Juárez et al. (46), vitamin E can shift in rumen PUFA biohydrogenation toward production of trans-11 C18:1 rather than trans-10 C18 through the action of *Butirivibrio fibrisolvens*, which is responsible for the hydrogenation of linoleic acid and linolenic acid to vaccenic acid (65). In contrast, Schäfers et al. (66) have reported that the use of the same quantity of vitamin E in the diet is unable to change the outflow of fatty acids from the rumen in Holstein dairy cows, as compared with dairy cows receiving a diet without vitamin E. Our results clearly showed that higher levels of PUFAs with higher concentrations of α-tocopherol in beef caused an increase in n-3 PUFA and CLA. Another possible explanation for the lower levels of n-3 and CLAs in the meat in group L may be that the oxidation of these FAs matched more in group L than LE. We hypothesize that vitamin E protected this FA in the LE group.

The beef n-6/n-3 FA ratio was higher in C than L and LE, thus demonstrating how dietary linseed decreases this ratio to the recommended value, lower than the threshold of 4.0, in beef meat (67). This result occurred for two reasons: the higher content of n-3 FA originating from extruded linseed integration and the decrease in n-6 concentration, in line with previous works (68, 69). The same results were found by Mordenti et al. (70) in fresh meat obtained from crossbred heifers fed with linseed.

EPA (20:5 n-3) and DHA (22:6 n-3) fatty acids are very important PUFAs recognized for their beneficial effects on the cardiovascular system and on the brain and visual system during fetal development and throughout life (71). The lower concentrations of SFAs, higher I-Harris index values (sum of EPA and DHA), and higher n-3PUFAs in groups L and LE than group C are important results in terms of the benefits to human health. However, the content of n-3 PUFA and CLAs was higher in LE than in L. These discrepancies probably occurred in response to the addition of vitamin E with linseed in the LE group different to the group that received only linseed. Indeed, vitamin E may counteract the shift in the biohydrogenation pathways by supporting the increase of the same fatty acid like CLA and same PUFA with a mechanism action that is not well-known (72). The peroxidation index was higher in LE than in L, despite the same concentration of linseed being used in the diet. This result was not surprising, given the higher value of long-chain fatty acids with double bonds in LE than L and C. The role of vitamin E was probably fundamental in preserving the concentrations of long-chain fatty acids over time to a greater extent than those in C and L. This result is consistent with findings reported by Jerónimo et al. (73), in which a higher PI in meat of lambs supplemented with oil was characterized by a higher content of PUFA. Indeed, in our study, the PI was calculated for meat after 9 days of storage and not on fresh meat. For this reason, the higher value of this index in LE can be considered favorable from a nutritional point of view.

The diet also affected CLA concentrations in beef meat in the three experimental groups, resulting in higher values in groups L and LE than C. These findings are consistent with results reported in the literature: diets rich in linoleic and linolenic acids are well-known to increase the levels of C18:1 t11 acid in meat as a product of rumen biohydrogenation, owing to stearoyl CoA desaturase, which converts vaccenic acids to the corresponding CLA 9c,11t isomer, the main CLA isomer, during the bio-hydrogenation process (74).

The role of vitamin E on the FAs composition of the beef meat was really fundamental. As explained before, vitamin E, on the one hand, protects beef meat PUFAs against peroxidation and, on the other hand, is able to affect the biohydrogenation pathways of dietary PUFAs in the rumen and consequently indirectly improving the CLA composition of meat products (75). However, other authors have not found any evidence of an effect on the fatty acid composition of beef meat by using a high dosage of vitamin E (50).

## Conclusion

Dietary supplementation with linseed significantly influenced the concentrations of PUFAs. Moreover, feeding a diet with linseed and vitamin E significantly affected the content of n-3 and CLA in meat. Our data also showed that EOs appear to protect unsaturated fatty acids against lipid oxidation for up to 9 days of storage. The use of vitamin E at 2.1 g/head/day in the diets of Marchigiana young bulls effectively decreased meat lipid oxidation for up to 9 days of storage in a modified atmosphere. The oxidative stability of the meat was affected by the use of dietary vitamin E and meat treatment with a blend of rosemary and oregano EOs, which enhanced the positive natural antioxidant effects during storage.

## Data Availability Statement

The raw data supporting the conclusions of this article will be made available by the authors, without undue reservation.

## Ethics Statement

Ethical review and approval was not required for the animal study because, this study was conducted within the normal breeding activities of the farm and the animals were slaughtered at the end of their productive cycle as indicated by the Protected Geographical Indication of White Beef of the Central Apennines. Written informed consent was obtained from the owners for the participation of their animals in this study.

## Author Contributions

ACM and MM performed the laboratory work. DC, LMEM, and LL performed data analysis. IF and MG wrote the manuscript. GV and AF assisted with editing the manuscript. All authors have read and agreed to the final version of the manuscript.

## Conflict of Interest

The authors declare that the research was conducted in the absence of any commercial or financial relationships that could be construed as a potential conflict of interest.
